# Antimicrobial susceptibility profiles of *Enterococcus* spp. isolates from domestic pigeons in Hungary in 2022

**DOI:** 10.3389/fvets.2025.1642910

**Published:** 2025-10-21

**Authors:** Ádám Kerek, Ábel Szabó, Ákos Jerzsele

**Affiliations:** ^1^Department of Pharmacology and Toxicology, University of Veterinary Medicine Budapest, Budapest, Hungary; ^2^National Laboratory of Infectious Animal Diseases, Antimicrobial Resistance, Veterinary Public Health and Food Chain Safety, University of Veterinary Medicine Budapest, Budapest, Hungary

**Keywords:** *Enterococcus*, antimicrobial resistance, minimum inhibitory concentration, MIC, pigeons, MDR

## Abstract

**Introduction:**

Antimicrobial resistance (AMR) in *Enterococcus* species is a growing global health concern, especially due to their resilience, gene transfer potential, and zoonotic implications. Pigeons (*Columba livia domestica*) may constitute overlooked reservoirs of antimicrobial-resistant Enterococcus spp.

**Methods:**

This study aimed to investigate the antimicrobial susceptibility profiles of *Enterococcus* isolates from pigeons in Hungary in 2022 and to assess the prevalence of multidrug-resistant (MDR), extensively drug-resistant (XDR), and pan-drug-resistant (PDR) phenotypes.

**Results:**

A total of 660 samples (oropharyngeal and cloacal swabs) were processed, from which 53 *Enterococcus* isolates (*Enterococcus faecium*, *Enterococcus faecalis*, *Enterococcus durans*, *Enterococcus mundtii*, *Enterococcus gallinarum*, *Enterococcus hirae* and *Enterococcus columbae*) were recovered as pure cultures and subjected to further analysis, representing 8.0% of the total samples and tested against 11 antibiotics using the broth microdilution method. Minimum inhibitory concentrations (MICs) were interpreted based on Clinical Laboratory Standards Institute (CLSI) and European Committee on Antimicrobial Susceptibility Testing (EUCAST) breakpoints or literature-based thresholds. Overall, 62.3% of isolates were MDR, 3.8% XDR, and no PDR. High resistance was observed for tylosin (81.1%), florfenicol (64.2%), and enrofloxacin (56.6%), while neomycin and potentiated sulfonamides showed full susceptibility. MIC90 values indicated retained efficacy for imipenem and neomycin. The decision tree identified florfenicol, enrofloxacin, and amoxicillin as key predictors of MDR. Monte Carlo simulation estimated an average MDR prevalence of 59.4% (95% CI: 50.0–69.0%).

**Discussion:**

The findings underscore pigeons’ potential role as environmental reservoirs of resistant *Enterococcus* isolates. This poses a concern for public and veterinary health under the One Health paradigm. These results support the urgent need for targeted antimicrobial surveillance in pigeons and further molecular investigations to characterize resistance determinants and assess potential transmission risks.

## 1 Introduction

Antimicrobial resistance (AMR) is an escalating global concern that jeopardizes the efficacy of antibiotics and significantly hinders the effective treatment of infectious diseases (1). Despite growing awareness of its impact, antimicrobial agents are still often administered without susceptibility testing, and sometimes even in cases where the underlying pathogen is not bacterial in nature (2). This is particularly true for animals kept for sport, such as racing pigeons, where owners may provide antibiotics prophylactically to maximize performance (2). Such misuse not only affects the treated animals but also contributes to environmental contamination, allowing antimicrobials to enter the systems of wild animals (3).

The role of wild birds, including feral pigeons — in the transmission of AMR is especially concerning, as they can act as carriers of zoonotic pathogens, including resistant bacterial strains. Several studies have confirmed that pigeons may serve as important reservoirs of AMR and can potentially facilitate interspecies transmission, including to humans (4). This is especially critical in the case of *Enterococcus* spp., which possess recognized zoonotic potential (5). In urban environments, feral pigeons live in exceptionally close proximity to humans, frequently sharing public spaces such as parks, plazas, and building structures. This daily cohabitation enhances the likelihood of environmental exposure to resistant bacteria, including through droppings and contaminated surfaces, thus raising the risk of zoonotic transmission (6, 7). Among bacterial diseases observed in pigeons, *Salmonella Typhimurium*–induced paratyphoid and *Escherichia coli* infections are the most common (8). However, infections caused by *Enterococcus* spp. are receiving increasing attention (5). Once considered harmless commensals, enterococci are now recognized as opportunistic pathogens capable of causing various conditions in pigeons (9), such as endocarditis and lameness associated with osteomyelitis (10). Bacteria of the genus *Enterococcus* are Gram-positive, facultatively anaerobic cocci, often found in the gastrointestinal tracts of humans and animals. They exhibit intrinsic resistance to several classes of antibiotics and are capable of acquiring additional resistance determinants through horizontal gene transfer, making them significant contributors to the global antimicrobial resistance crisis (11).

The emergence of multidrug-resistant (MDR) *Enterococcus* strains has become more prevalent, particularly in nosocomial settings (12). These strains can rapidly colonize the human gastrointestinal tract following antibiotic treatment and displace commensal microbiota (13, 14). The widespread use of antibiotics has contributed to the proliferation of resistant enterococci, which are now among the most common nosocomial pathogens (12). MDR strains are defined as those resistant to at least one agent in three or more antimicrobial categories; extensively drug-resistant (XDR) strains are resistant to all but two or fewer categories; and pan-drug-resistant (PDR) strains are non-susceptible to all agents in all antimicrobial categories tested (15).

Of the approximately 35 known *Enterococcus* species (16) the most frequently isolated from pigeons include *E. columbae* (17), *E. caecorum*, and *E. faecalis* (18). These species are known reservoirs of resistance genes (19), and as such play a key role in the dissemination of AMR (20). Moreover, the persistence of enterococci on inanimate surfaces and medical equipment — outside the gastrointestinal tract — facilitates indirect and direct transmission, including by healthcare personnel (12). Studies have identified several *Enterococcus* species in pigeons, with *E. columbae*, *E. hirae*, *E. faecalis*, and *E. faecium* being the most prevalent. Notably, *E. columbae* is considered host-specific to pigeons and often dominates their enterococcal population. These species exhibit varying patterns of antimicrobial resistance. For instance, *E. columbae* has shown significant resistance to chloramphenicol, while *E. faecalis* and *E. faecium* are frequently resistant to tetracycline and erythromycin. The presence of such resistant strains in pigeons underscores the importance of species-level identification in antimicrobial resistance studies, as it provides insights into potential reservoirs and transmission pathways of resistant bacteria (9).

The use of antimicrobials for growth promotion was fully banned in the European Union in 2006 (21). However, while this regulation has contributed to curbing the spread of AMR, resistance in *Enterococcus* spp. remains a serious concern (22, 23). Antibiotics commonly used to treat *Enterococcus*-associated infections in veterinary practice include amoxicillin, cephalosporins, doxycycline, enrofloxacin, spectinomycin, tylosin, and potentiated sulfonamides (24).

To mitigate antibiotic usage, increasing emphasis is placed on alternative strategies that may fully or partially replace antimicrobials or complement their use (25–32). Additionally, strict adherence to biosecurity measures (33), and antibiotic selection based on pharmacokinetic and pharmacodynamic principles, are essential tools in the fight against AMR (24, 34).

The aim of the present study was to determine the antimicrobial susceptibility profiles of *Enterococcus* spp. isolates recovered from domestic pigeons in Hungary in 2022. The findings contribute to a more nuanced understanding of the role of *Enterococcus* spp. in AMR dissemination and may inform future veterinary and public health strategies.

## 2 Results

### 2.1 Bacterial species composition and source characteristics

**Figure 1:**
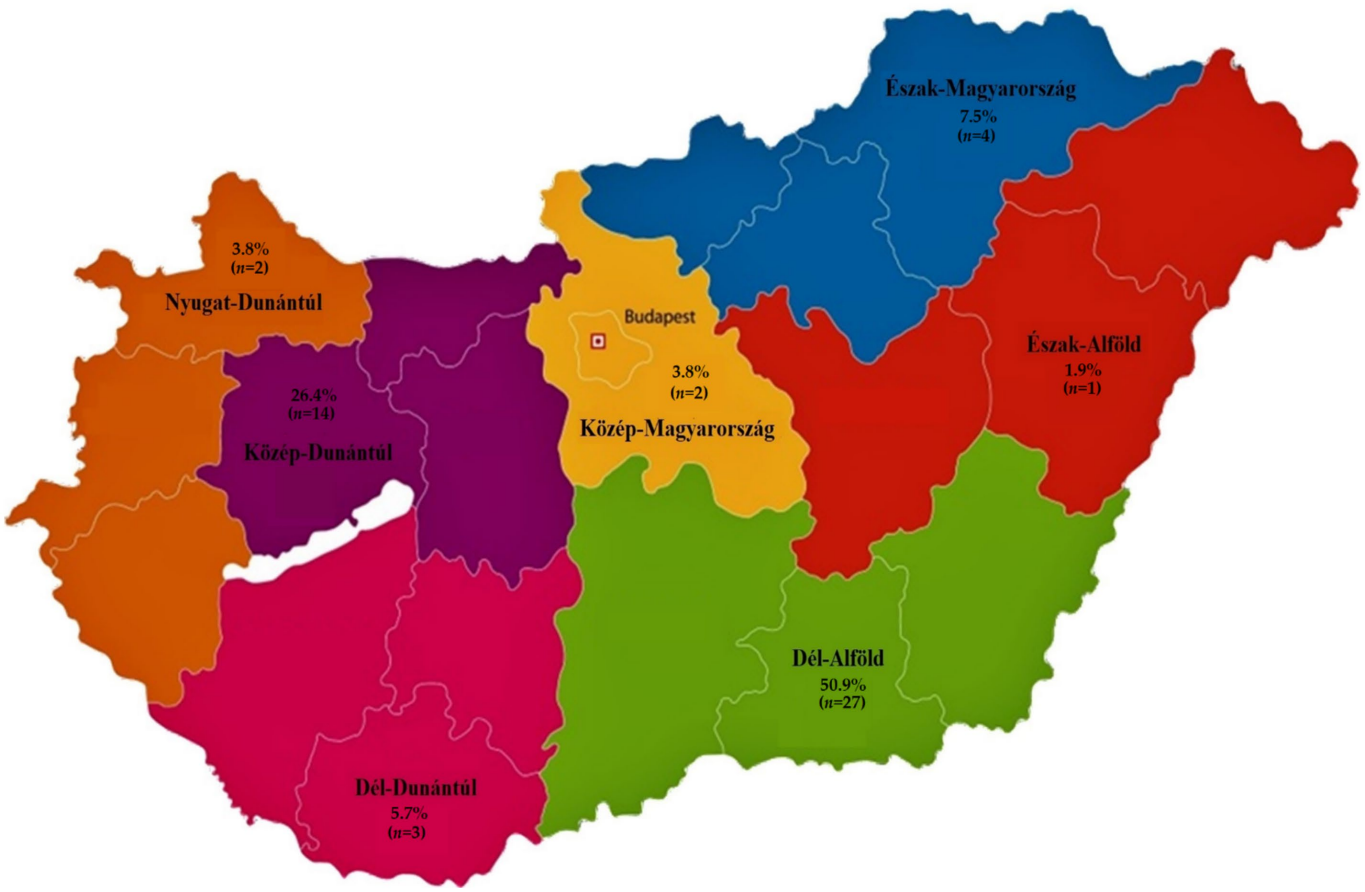
Distribution of *Enterococcus* isolates (*n* = 53) isolated from pigeons across Hungary.

In this study, a total of 53 *Enterococcus* isolates isolated from pigeons were subjected to antimicrobial susceptibility testing. Out of the total 53 samples, 15 were collected from the tracheal region and 38 from the cloacal region. According to MALDI-TOF identification, the *Enterococcus* isolates consisted of 13 *Enterococcus faecium*, 21 *Enterococcus faecalis*, 7 *Enterococcus durans*, 1 *Enterococcus mundtii*, 4 *Enterococcus gallinarum*, 2 *Enterococcus hirae* and 5 *Enterococcus columbae* (Supplementary Table 1). More than half of the isolates (50.9%) originated from the Dél-Alföld region of Hungary. The majority were recovered from homing pigeons (39.6%), with most birds classified as juvenile pigeons (as defined by <6 weeks of age) accounted for 39.6% of the sampled population and 58.5% of owners maintained medium-sized flocks (51–100 birds). The regional distribution of the isolates is presented in Figure 1. Descriptive data on sample origin, age distribution, flock size by region, and the proportion of MDR isolates are summarized in Supplementary Tables S2–S4.

### 2.2 MIC distribution and susceptibility profiles

Following MIC determination, a comprehensive frequency table was generated (Table 1), which includes both MIC₅₀ and MIC₉₀ values for each antibiotic tested. This table provides a quantitative overview of the antimicrobial susceptibility distribution across the entire isolates population and facilitates interpretation by aligning the values with CLSI breakpoints and EUCAST epidemiological cut-off values (ECOFFs).

**Table 1:** Frequency distribution table of minimum inhibitory concentrations (MICs) for *Enterococcus* isolates (*n* = 53) from pigeons, tested against antibiotics with established clinical breakpoints.

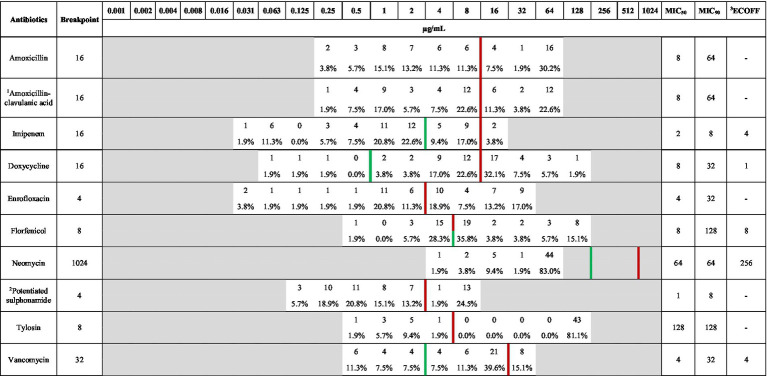

The upper row represents the frequency values, while the lower row indicates the corresponding percentage. Vertical red lines denote clinical breakpoints, while vertical green lines represent the epidemiological cutoff values (ECOFF) defined by the European Committee on Antimicrobial Susceptibility Testing (EUCAST).

^1^Ratio 2:1.

^2^Trimethoprim and sulfamethoxazole in a 19:1 ratio.

^3^Epidemiological cutoff values (ECOFF) defined by the European Committee on Antimicrobial Susceptibility Testing (EUCAST).

MIC_90_ values indicated relatively low resistance to imipenem (8 μg/mL). While neomycin also showed a low MIC90 (64 μg/mL), the absence of an established clinical breakpoint for *Enterococcus* spp. limits interpretation. In contrast, a more variable susceptibility pattern was observed for other agents, where at least 50% of isolates were susceptible to amoxicillin, amoxicillin-clavulanate, doxycycline, potentiated sulfonamide, and vancomycin, based on MIC₅₀ values.

Importantly, comparison with ECOFFs revealed that 90% or more of the isolates were wild-type for neomycin, while imipenem also showed a wild-type profile in over 50% of cases. These findings not only corroborate the results from categorical resistance classifications but also provide a finer resolution of MIC trends within the population.

Table 1 was included to offer a detailed antimicrobial profile, identify potential therapeutic options, and highlight the antibiotics that may still be effective against commensal isolates. The inclusion of this table also allows comparison with future surveillance datasets.

Detailed MIC data at the isolate level are made available in the Supplementary material (file to enhance) transparency and reproducibility.

**Figure 2:**
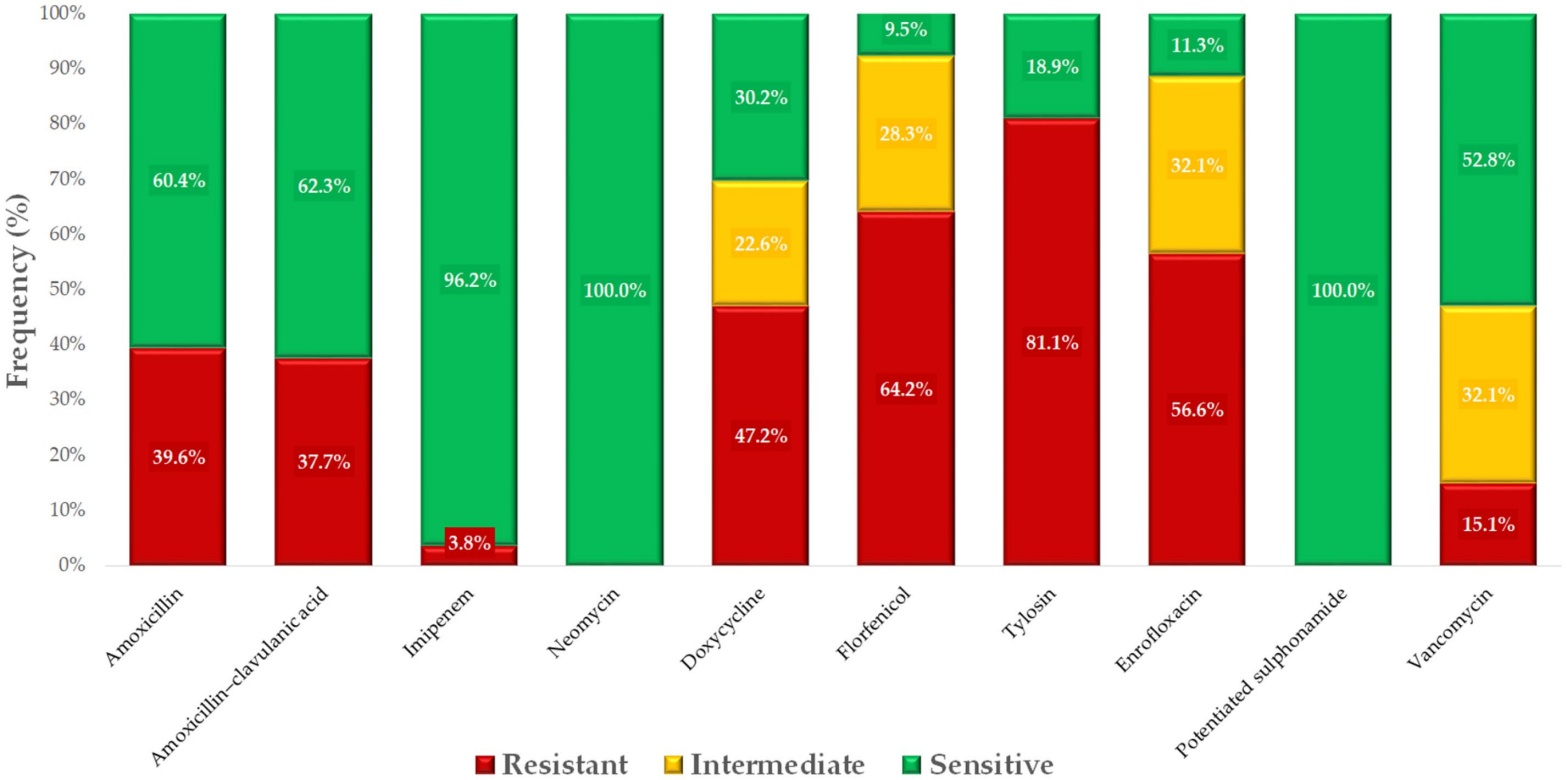
Antibiogram of *Enterococcus* isolates (*n* = 53) isolated from pigeons, based on clinical breakpoints for key antimicrobials.

Figure 2 presents the distribution of *Enterococcus* isolates classified as resistant or susceptible to each antimicrobial agent based on established CLSI breakpoints. This figure was included to visually summarize resistance prevalence across the tested antibiotic panel and to identify key agents with high or low effectiveness within the population.

### 2.3 Phenotypic resistance prevalence and multidrug resistance status

**Figure 3:**
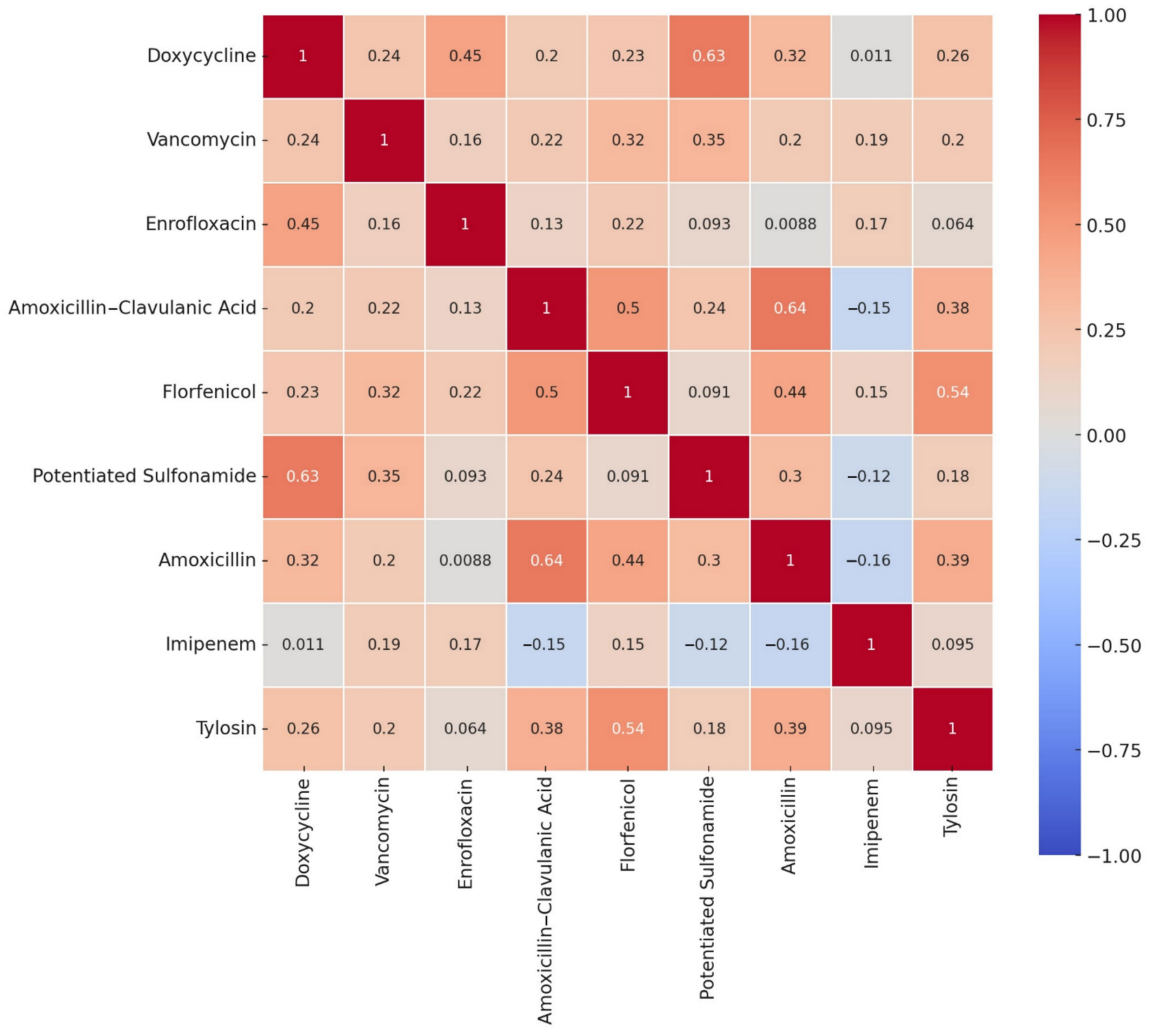
Correlation heatmap of antibiotic resistance profiles for *Enterococcus* isolates (*n* = 53) from pigeons. The heatmap depicts the strength and direction of the correlations (*r*-values) between antibiotics, with red tones indicating positive correlations and blue tones indicating negative ones. Strong positive correlations were observed between potentiated sulfonamide and doxycycline (0.63), amoxicillin and amoxicillin–clavulanic acid (0.64), and florfenicol and tylosin (0.54), suggesting potential co-selection or co-resistance mechanisms. These relationships may reflect shared resistance determinants, co-located resistance genes on mobile elements, or common patterns of antimicrobial usage in avian hosts. Weak or negligible correlations were observed with imipenem, which behaved largely independently of the other agents. This visual analysis provides insights into the non-random distribution of resistance traits and informs subsequent modeling steps such as decision tree analysis and network mapping.

Pearson correlation analysis was employed to explore potential associations between resistances to different antibiotics, offering insights into co-selection patterns and shared resistance mechanisms. The corresponding heatmap (Figure 3) was selected for its ability to visually represent the strength and direction of these relationships. Notably, strong positive correlations were detected between amoxicillin and amoxicillin-clavulanate (0.64), doxycycline and potentiated sulfonamides (0.63), and florfenicol and tylosin (0.54).

Based on phenotypic profiles, 62.3% of isolates met the criteria for MDR, and 3.8% classified as XDR, according to established definitions. No PDR isolates were observed.

### 2.4 Clustering analysis of resistance profiles

**Figure 4:**
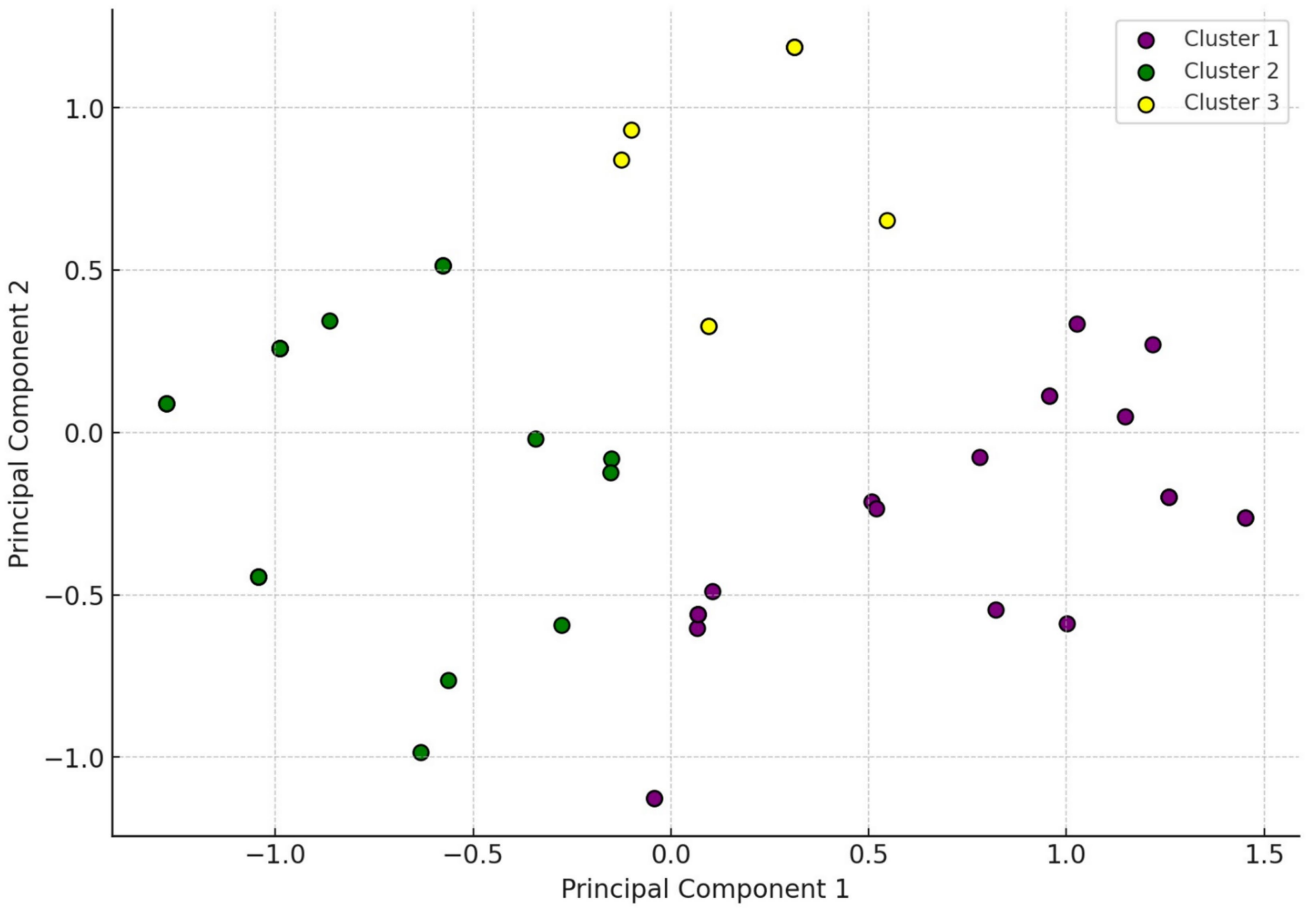
Hierarchical cluster analysis of *Enterococcus* isolates (*n* = 53). The two-dimensional scatter plot visualizes the distribution of isolates along the first two principal components (PC1 = 38.6%, PC2 = 21.7%), which together account for 60.3% of the variance in resistance data. K-means clustering (*k* = 3) identified three distinct clusters: Cluster 1 (purple), characterized by high-level resistance to doxycycline; Cluster 2 (green), dominated by enrofloxacin resistance; and Cluster 3 (yellow), showing elevated resistance to amoxicillin–clavulanic acid. The spatial separation of clusters highlights underlying heterogeneity in resistance phenotypes, potentially linked to differences in antibiotic exposure, genetic backgrounds, or environmental factors. This unsupervised analysis supports the existence of biologically relevant subgroups within the *Enterococcus* population, offering insights into the distribution of MDR profiles across isolates. The clustering results also guide interpretation in subsequent decision tree and network graph analyses by illustrating distinct resistance pattern groupings.

Cluster analysis was conducted to identify natural groupings of isolates based on their resistance profiles, helping to uncover patterns not immediately evident through individual antibiotic comparisons. The resulting K-means clustering identified three distinct clusters (Figure 4), each characterized by dominant resistance phenotypes that suggest differing selection pressures or genetic backgrounds. Cluster 1 was primarily defined by high resistance to doxycycline, suggesting potential overuse or cross-resistance with other tetracyclines. Cluster 2 was dominated by enrofloxacin resistance, consistent with the widespread use of fluoroquinolones in intensive poultry production. Cluster 3 was most strongly associated with amoxicillin-clavulanate resistance.

### 2.5 Co-resistance network analysis

The inclusion of this analysis contributes to the study’s findings by demonstrating heterogeneity within the sample set, reinforcing the notion that resistance dynamics may differ between production systems or bacterial lineages.

**Figure 5:**
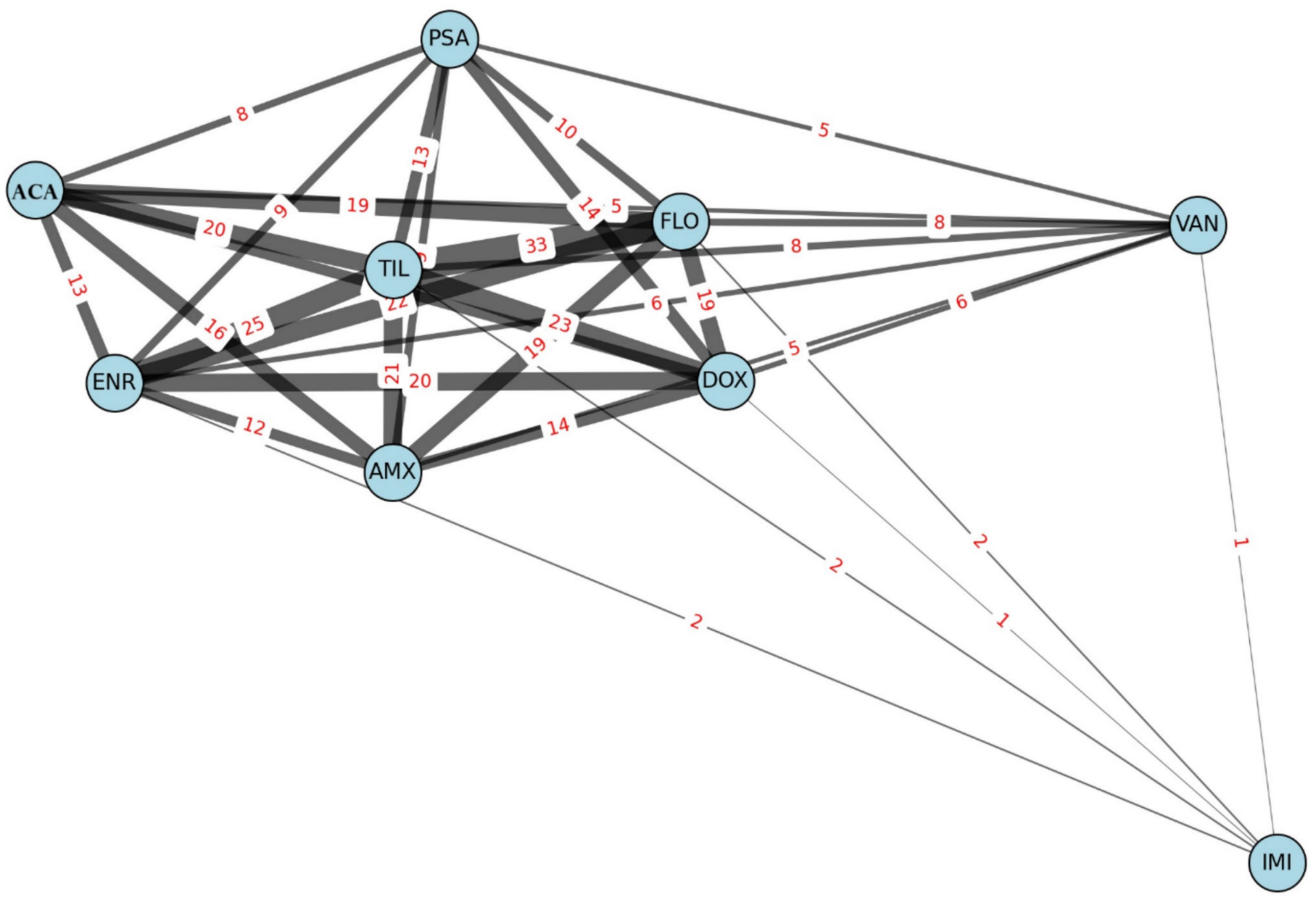
Network graph illustrating co-resistance patterns among *Enterococcus* isolates (*n* = 53) based on antibiotic susceptibility profiles. AMX, amoxicillin; ACA, amoxicillin-clavulanic acid; DOX, doxycycline; ENR, enrofloxacin; FLO, florfenicol; IMI, imipenem; PSA, potentiated sulfonamide; TIL, tylosin; VAN, vancomycin.

Network graph analysis was employed to examine co-resistance patterns among antimicrobials, offering a visual representation of how frequently resistance co-occurred across isolates (Figure 5). This approach helps to identify potential co-selection mechanisms or linked resistance determinants that may be transmitted together.

Edge thickness in the graph reflects the frequency of co-resistance between two antibiotics, providing an intuitive map of resistance clustering. Tylosin emerged as a central node, showing the highest number of co-resistances, particularly with florfenicol (*n* = 33), enrofloxacin (*n* = 25), and doxycycline (*n* = 23). These frequent pairings suggest shared resistance pathways or common usage in poultry production that may drive joint selection.

Conversely, imipenem- and vancomycin-resistant isolates appeared as peripheral or isolated nodes, indicating that resistance to these agents is rare and does not typically co-occur with resistance to other compounds.

### 2.6 Decision tree model for MDR prediction

The decision tree, which consisted of four hierarchical levels, effectively distinguished MDR from non-MDR isolates. This supervised machine learning method enables the stepwise stratification of isolates based on their resistance profiles, helping to pinpoint variables most predictive of MDR.

The tree grew to a maximum depth of four, balancing model interpretability and classification accuracy. Among the 15 tested antibiotics, resistance to florfenicol, potentiated sulfonamide, enrofloxacin, and amoxicillin emerged as the most discriminative features, sequentially splitting the dataset into MDR and non-MDR subsets. However, a 10-fold cross-validation yielded an overall accuracy of 75%, precision of 72%, and recall of 78%, suggesting reasonable internal consistency despite the sample size limitation.

This finding suggests that resistance to these compounds — particularly when occurring in combination — strongly correlates with broader resistance phenotypes. The model’s predictive structure offers practical insight into field diagnostics: identifying resistance to just a few of these antibiotics may serve as an early warning indicator for MDR emergence.

### 2.7 Monte Carlo simulation for MDR benchmarking

**Figure 6:**
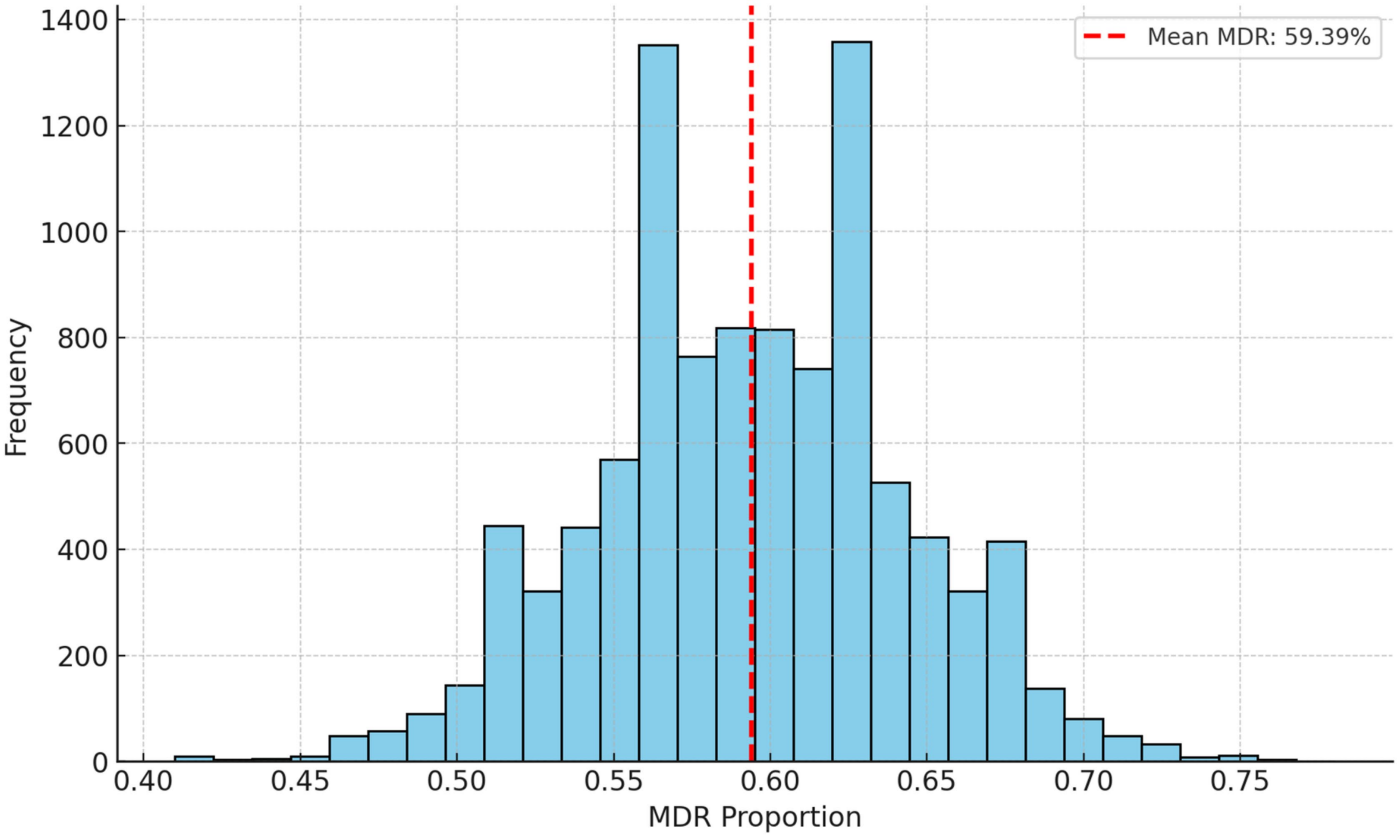
Monte Carlo simulation results for estimating the prevalence of multidrug resistance in *Enterococcus* isolates. The mean multidrug-resistance (MDR) rate was 59.39% over 10,000 iterations. The simulation was conducted using a frequentist approach, with random sampling based on the observed MDR rate to assess variability. No Bayesian priors or probabilistic modeling were applied.

To complement the empirical findings and assess the expected baseline of MDR, a Monte Carlo simulation was performed using 10,000 iterations (Figure 6). This stochastic modeling technique enabled the estimation of MDR prevalence under conditions of random resistance distribution, while preserving the real dataset’s size and antibiotic count.

The simulation yielded an average MDR rate of 59.39%, with a normal distribution curve reflecting consistent simulation behavior. The 95% confidence interval ranged from 50.0 to 69.0%, which provides a robust benchmark against which the observed MDR rate (62.3%) can be compared.

Figure 6 thus serves as a statistical validation of the MDR rates, reinforcing the conclusion that strategic interventions are needed to mitigate further selection and spread.

### 2.8 Comparison with human AMR datasets

**Figure 7:**
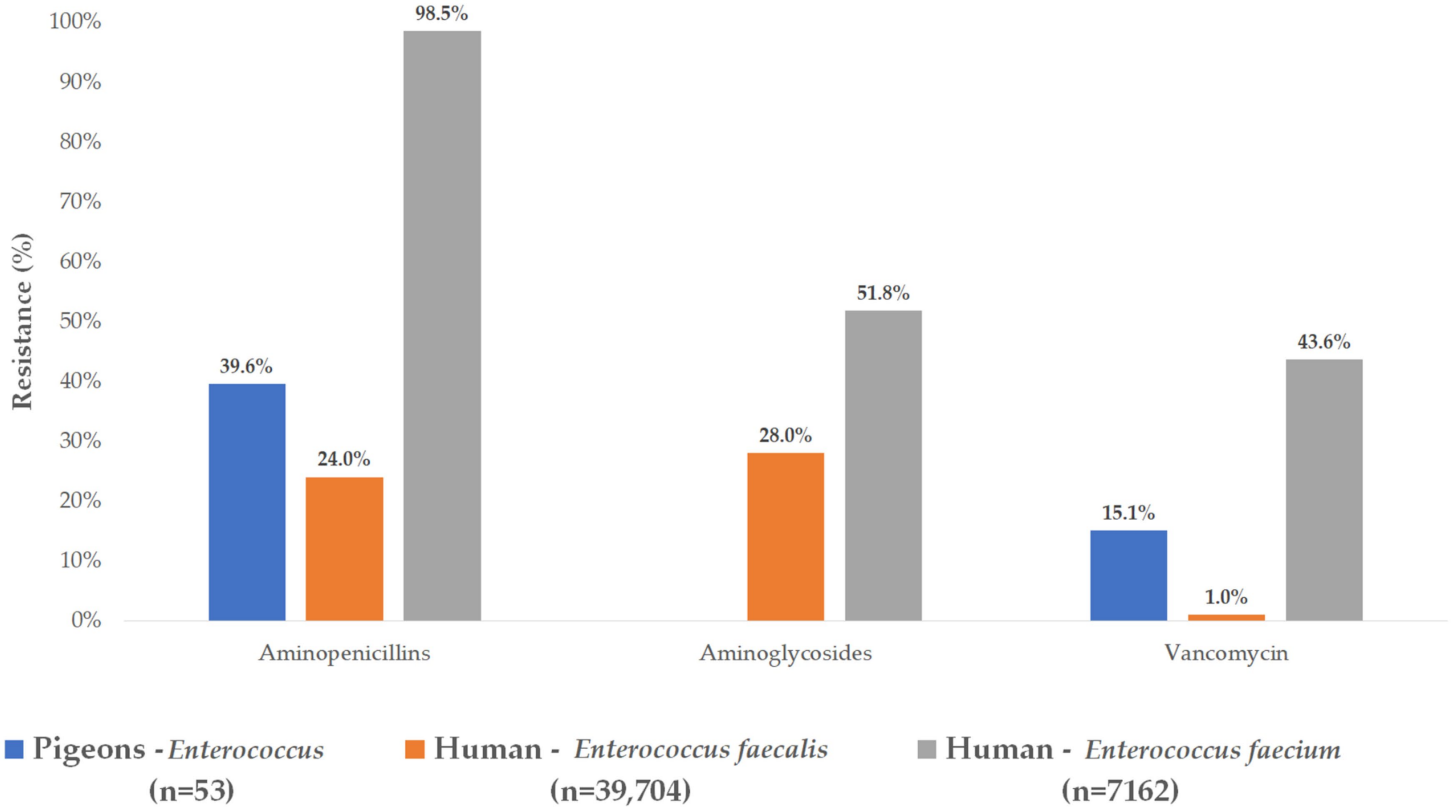
Comparative analysis of resistance levels in *Enterococcus* isolates isolated from pigeons and those reported in human clinical data.

We also compared our findings with publicly available human antimicrobial resistance data to assess potential overlaps and One Health relevance (Figure 7). For aminopenicillins, the resistance profile of our isolates resembled that of *E. faecalis* from human clinical settings, which typically shows low resistance levels. This similarity may indicate shared susceptibility patterns between poultry- and human-associated isolates of this species.

However, a stark contrast was observed with *E. faecium*, where nearly all human isolates (98.5%) displayed aminopenicillin resistance, highlighting the species-specific nature of resistance development. This divergence suggests that *E. faecium* poses a higher therapeutic challenge in clinical practice and may reflect distinct evolutionary or usage pressures in human versus veterinary contexts.

Notably, none of the poultry-derived isolates showed resistance to aminoglycosides, a finding that diverges from certain human clinical isolates, where aminoglycoside resistance is more prevalent.

Vancomycin resistance, a major concern for both animal and human health, was identified in 15.1% of our poultry isolates. When species-level human data were considered, only 1.0% of *E. faecalis* isolates were resistant, whereas resistance was significantly higher (43.6%) among human *E. faecium* isolates.

## 3 Discussion

Our findings reveal a substantial burden of antimicrobial resistance among *Enterococcus* isolates derived from pigeons in Hungary, with more than 60% of strains meeting the criteria for MDR. The detection of Enterococcus species in both tracheal and cloacal swabs indicates that these bacteria can colonize multiple anatomical sites in poultry, including both the respiratory and intestinal tracts. This dual presence may suggest a wider ecological adaptability and potential for dissemination within the host and the farm environment. Resistance was particularly pronounced against tylosin, florfenicol, and enrofloxacin, highlighting the possible selective pressures exerted by these commonly used agents in avian medicine. In addition to characterizing resistance rates, we employed a suite of statistical and computational approaches — including correlation analysis, clustering, network graphing, decision tree modeling, and Monte Carlo simulation — to uncover co-resistance patterns, infer underlying resistance mechanisms, and compare observed resistance distributions with simulated random expectations. This integrated analytical framework enabled a more robust interpretation of the resistance landscape and suggested that resistance patterns are shaped by non-random, potentially management-related selective forces rather than stochastic variation alone, underscoring the need for targeted stewardship interventions in avian health.

Nationwide, a total of 53 *Enterococcus* spp. isolates were included in the resistance profiling. Among MIC_50_ values, only imipenem (2 μg/mL) exhibited retained susceptibility, while none of the agents showed susceptibility at the MIC_90_ level.

It is important to differentiate between MIC values and ECOFFs, as the latter distinguish wild-type from non-wild-type populations. Identifying non-wild-type isolates is essential for detecting emerging resistance before it reaches clinical significance. This is particularly relevant for monitoring low-level resistance development and assessing selective pressure in non-clinical environments (35).

In a Czech study analyzing 131 *Enterococcus* isolates, the highest resistance rate (20%) was reported against tetracyclines, with 9% for gentamicin and 3% for vancomycin (36). In contrast, our findings revealed considerably higher resistance rates: 94% for doxycycline and 15.1% for vancomycin. While Butaye et al. reported no resistance to vancomycin, they identified 14% resistance to enrofloxacin and 74% to tylosin (37), whereas our study revealed 91 and 81%, respectively. In Brazil, 120 *Enterococcus* isolates from urban pigeons showed 100% resistance to vancomycin (38), whereas in our study, 66% of isolates were resistant. In Poland, among 145 isolates, 0.7% showed resistance to amoxicillin-clavulanic acid, 80% to enrofloxacin, 73.1% to doxycycline, and 19.3% to vancomycin (9). Our corresponding rates were 58, 91, 94, and 66%, respectively.

Dolka et al. examined cloacal swabs from racing pigeons and found that 93.1% of the *Enterococcus* isolates were resistant to at least one antibiotic, and 29% were resistant to four agents. Tetracyclines showed the highest resistance, followed by enrofloxacin and doxycycline (9), consistent with our data indicating a high prevalence of MDR.

Radimersky et al. reported lower resistance rates in feral pigeons, especially for tetracyclines (38). This discrepancy is likely due to differences in antibiotic exposure between domestic and wild pigeon populations. A recent study by Garcia-Llorens et al. reported an MDR prevalence of 86% in *Enterococcus* isolates from laying hens in Zambia, which exceeds the rate found in our pigeon isolates. This suggests that antibiotic use practices in the poultry industry may strongly influence AMR development (39).

Pigeons are frequently in close contact with humans and other animals, offering opportunities for AMR transmission across species. Furthermore, their flight capabilities facilitate the geographical spread of resistant isolates. This highlights the need for continuous surveillance of pigeon populations as part of a broader AMR monitoring strategy and One Health approach.

The strong correlations observed — such as between tylosin and florfenicol — may indicate co-selection or co-resistance mechanisms, such as the presence of linked resistance genes on mobile genetic elements. For instance, resistance genes like *ermB* and *fexA*, frequently associated with macrolide and phenicol resistance, respectively, can be co-located on plasmids or transposons, contributing to their joint selection (40).

The central role of tylosin in the network graph (Figure 4) highlights its significant co-resistance potential. Tylosin’s frequent use in veterinary settings may exert substantial selection pressure, promoting the co-occurrence of resistance to other agents. Its high number of edges suggests that tylosin resistance often co-exists with resistance to other classes, thus acting as a proxy for multidrug resistance.

The elevated resistance rates observed in pigeon-derived *Enterococcus* isolates to several antibiotics (notably amoxicillin, doxycycline, tylosin, florfenicol, and erythromycin) may be attributable to multiple environmental and anthropogenic factors. Domestic pigeons are often kept in close proximity to humans, where they may be unintentionally exposed to antimicrobials through contaminated water, feed, or via cross-contact with medicated poultry species. One possible contributing factor is the use of leftover or unregulated feed originating from poultry farms, which may contain subtherapeutic residues of antibiotics. This selective pressure could drive the persistence of resistant strains in pigeon populations. Furthermore, erythromycin and tylosin are frequently used in veterinary medicine, particularly in poultry, and may exert indirect selection pressure on synanthropic avian species through environmental dissemination. The role of unmonitored antibiotic exposure via contaminated environments such as urban pigeon habitats should not be overlooked, as it can sustain and amplify resistant bacterial populations over time (41).

The results indicate that neomycin and potentiated sulfonamide retained full sensitive. These findings suggest that these agents may still serve as effective options against commensal *Enterococcus* isolates in poultry settings. Imipenem and vancomycin also showed relatively low resistance rates (3.8 and 15.1%, respectively), which is particularly relevant given their critical role in human medicine. These data may support the notion that selective pressure from veterinary use is limited for these drugs, at least within the studied region. Conversely, extremely high resistance rates were observed for tylosin (81.1%), florfenicol (64.2%), and enrofloxacin (56.6%), raising concerns about their widespread use in poultry production and the potential selection of resistant isolates. The inclusion of these data in a comparative figure format helps clarify which antibiotics are driving resistance and supports the rationale for more targeted use and stewardship. These visual insights contribute directly to the study’s conclusions by identifying specific antibiotics that are most affected by resistance trends in the Dél-Dunántúl region.

The proximity of the high MDR observed value to the upper end of the simulated range suggests a non-random distribution of resistance traits, likely influenced by external selective pressures such as antibiotic usage practices. This supports the hypothesis that AMR development in the examined poultry populations is not purely stochastic but driven by environmental and management factors.

The relatively low number of isolates analyzed (*n* = 53) is a key limitation that constrains the statistical power of the applied models and restricts the extrapolation of findings. While confidence intervals or model performance metrics (e.g., accuracy, sensitivity, specificity) were not calculated due to sample size constraints, the decision tree and simulation outputs should be interpreted as exploratory tools providing useful hypotheses for future studies with larger datasets.

One important limitation of this study is the absence of molecular data regarding antimicrobial resistance genes. While phenotypic susceptibility testing provides essential insights into the resistance profiles of the isolates, it does not reveal the underlying genetic determinants. Without information on the presence or distribution of specific resistance genes, it is not possible to assess the potential for horizontal gene transfer, co-resistance, or the presence of mobile genetic elements such as plasmids or transposons. Furthermore, the lack of genetic data hinders the ability to distinguish between acquired and intrinsic resistance mechanisms. Future studies should incorporate molecular approaches such as PCR or whole genome sequencing to confirm and complement the phenotypic findings, allowing for a deeper understanding of the genetic context of resistance and supporting more targeted antimicrobial stewardship strategies.

These results suggest that pigeons may harbor multidrug-resistant *Enterococcus* spp.; however, further studies are needed to determine their potential role in zoonotic transmission.

## 4 Materials and methods

### 4.1 The origin of the strains

The *Enterococcus* isolates (*n* = 53) analyzed in this study were recovered from a total of 660 oropharyngeal and cloacal swab samples collected in 2022 with the assistance of veterinary practitioners attending healthy domestic pigeons (*Columba livia domestica*), maintained in private lofts for sport, for meat or ornamental purposes. This represents a recovery rate of 8.0%. The term “domestic” here refers to birds bred and kept under human care, as opposed to feral or wild-living pigeons. Sampling was performed on a random basis, with the specific aim of maximizing geographic coverage across Hungary to enhance the representativeness of the collected isolates. Oropharyngeal and cloacal swab samples were collected during routine diagnostic examinations. The samples were obtained from pigeon populations located in Észak-Magyarország (*n* = 3), Észak-Alföld (*n* = 2), Dél-Alföld (*n* = 8), Közép-Magyarország (*n* = 5), Közép-Dunántúl (*n* = 1), Dél-Dunántúl (*n =* 1), and Nyugat-Dunántúl (*n* = 2). The isolates were obtained as pure cultures derived from the collected oropharyngeal and cloacal samples. Sampling was performed using Amies-type (carbon-free, aluminum shaft) swabs (Biolab Zrt., Budapest, Hungary). We spread the samples on m-*Enterococcus* modified agar (Merck KGaA, Darmstadt, Germany) for the identification of the *Enterococcus* genus, following the manufacturer’s specifications, which indicate that only *Enterococcus* species exhibit growth on this medium, with colony colors ranging from light pink to red. The species identification of the strains was determined using MALDI-TOF mass spectrometry (Flextra-LAB Kft., Budapest, Hungary) and Biotyper software version 12.0 (Bruker Daltonics GmbH, Bremen, Germany, 2024) (42). The isolates were stored at −80 °C using the Microbank™ cryopreservation system (Pro-Lab Diagnostics, Richmond Hill, Canada).

Human antimicrobial resistance data were provided by the National Public Health Center of Hungary. Resistance to ampicillin was used as the comparator in human cases, while amoxicillin was used for veterinary isolates. For third-generation cephalosporins, ceftriaxone was selected for comparison. Aminoglycoside resistance data were aggregated across gentamicin, tobramycin, and amikacin; additional specific data for neomycin were also available. Fluoroquinolone resistance was assessed as a group in human isolates, whereas enrofloxacin was evaluated separately for veterinary samples. Both national and region-specific human data were shared in Excel format with official authorization from the Chief Medical Officer and included percentage resistance rates.

### 4.2 Minimum inhibitory concentration (MIC) determination

Phenotypic resistance was evaluated by determining MIC values according to the guidelines of the Clinical Laboratory Standards Institute (CLSI) (43). Breakpoints were interpreted using CLSI standards [35], and compared with epidemiological cut-off values (ECOFFs) published by the European Committee on Antimicrobial Susceptibility Testing (EUCAST) (44). The choice to apply CLSI breakpoints was based on their widespread usage in veterinary antimicrobial susceptibility testing and the broader availability of CLSI interpretive criteria for the antimicrobials tested. While EUCAST clinical breakpoints are increasingly adopted in European human healthcare settings, CLSI remains a practical and accepted standard in animal studies. EUCAST ECOFFs were included to complement the clinical interpretation with an epidemiological assessment, distinguishing wild-type from non-wild-type isolates and providing a broader perspective on emerging resistance trends. This dual approach allows for both clinically and epidemiologically relevant interpretations, and has been applied in previous veterinary AMR surveillance studies (45, 46).

In the case of neomycin, no CLSI or EUCAST breakpoint is available for *Enterococcus* spp., thus we referred to a meat-product–based threshold of 1,024 μg/mL reported in the literature (47). Similarly, for tylosin, an 8 μg/mL breakpoint was adopted from published sources (48).

The bacterial isolates stored at −80 °C were revived 1 day before testing by suspending them in 3 mL of cation-adjusted Mueller-Hinton broth (CAMHB; Biolab Zrt, Budapest, Hungary), followed by incubation at 37 °C for 18–24 h. MIC testing was performed using 96-well microtiter plates (VWR International, LLC., Debrecen, Hungary). Except for the first column, each well was filled with 90 μL of CAMHB. Stock solutions of 1,024 μg/mL of each tested antimicrobial agent (Merck KGaA, Darmstadt, Germany) were prepared according to CLSI protocols (49).

Amoxicillin and amoxicillin-clavulanate were dissolved in phosphate buffer (2:1 ratio; pH 7.2, 0.01 mol/L), while imipenem was dissolved in phosphate buffer at pH 6. Doxycycline, neomycin, tylosin, and vancomycin were dissolved in distilled water. For potentiated sulfonamides (trimethoprim-sulfamethoxazole, 1:19 ratio), sulfamethoxazole was dissolved in hot water with a few drops of 2.5 mol/L NaOH, and trimethoprim was dissolved in 0.05 mol/L HCl-containing distilled water. Enrofloxacin was dissolved in distilled water with a few drops of 1 mol/L NaOH, and florfenicol was dissolved in 95% ethanol diluted with distilled water. All antimicrobial agents used in this study were obtained from Sigma-Aldrich (Merck KGaA, Darmstadt, Germany).

A 180 μL aliquot from a 512 μg/mL stock solution (diluted 1:1 with CAMHB) was pipetted into the first well, followed by a two-fold serial dilution across columns. After the 10th column, excess volume was discarded to maintain 90 μL per well. The bacterial inoculum was adjusted to 0.5 McFarland standard using a nephelometer (ThermoFisher Scientific, Budapest, Hungary), and 10 μL was added to each well starting from column 11 and proceeding backward (43).

MIC values were read using the Sensititre™ SWIN™ system (ThermoFisher Scientific, Budapest, Hungary) and interpreted with VIZION System Software v3.4 (ThermoFisher Scientific, Budapest, Hungary, 2024). MICs were determined once per isolate, following CLSI guidelines, as is standard practice in large-scale antimicrobial surveillance studies. The reference quality control strain used was *E. faecalis* ATCC 29212. This strain is recommended by CLSI guidelines for antimicrobial susceptibility testing of *Enterococcus* spp. and was employed to ensure the accuracy and reliability of MIC determinations throughout the study.

### 4.3 Statistical analysis

All statistical analyses were performed using R version 4.2.2 in the RStudio environment (50). The Shapiro–Wilk test was used to assess normality. For non-normally distributed data, non-parametric tests were applied. Differences in antimicrobial resistance across categories were evaluated using the Kruskal–Wallis test (51), which does not assume normality and is suitable for comparing medians across multiple groups. *Post hoc* pairwise comparisons were carried out using Mann–Whitney U tests (52), and t-tests, with Bonferroni correction applied to adjust for inflated *p*-values due to multiple comparisons (53). The potential risk of increased Type II error (false negatives) associated with the Bonferroni correction was taken into account.

Correlation analysis between antimicrobial agents was visualized via heatmaps using the *corrplot* (v0.92) and *pheatmap* (v1.0.12) R packages. Hierarchical clustering was performed using the *cluster* (v2.1.4) package, visualized with *factoextra* (v1.0.7) and *dendextend* (v1.16.0).

To identify patterns of co-resistance, network graph analysis was conducted using the *igraph* (v1.3.5) and *ggraph* (v2.1.0) packages. In the network graph, each node represented a specific antimicrobial agent, and edges were drawn based on pairwise co-resistance patterns derived from binary resistance data (resistant = 1, susceptible = 0) across isolates. The thickness of edges corresponded to the number of isolates exhibiting simultaneous resistance to both agents, highlighting the frequency of co-occurrence. Overall, the network graph complements the statistical analyses by revealing structural patterns of resistance interaction, further substantiating the selective pressures shaping the resistance landscape in the studied region.

For MDR isolates prediction, a decision tree model was built using *rpart* (v4.1.16), evaluated with *caret* (v6.0.93), and visualized using *rpart.plot* (v3.1.2). Given the relatively small sample size, the decision tree analysis was used as an exploratory tool to identify patterns rather than to produce definitive predictive metrics. In the decision tree, the input variables were binary resistance profiles (0 or 1) for each antibiotic, and the target variable was the classification of isolates as MDR or non-MDR based on established criteria. The model identified which antibiotics’ resistance status was most predictive of MDR and used sequential splits to stratify isolates accordingly.

Monte Carlo simulations were employed to estimate the prevalence of MDR isolates using 10,000 bootstrap iterations. The *boot* (v1.3.28), *dplyr* (v1.1.0), and *ggplot2* (v3.4.0) packages were used for resampling, aggregation, statistical summary, and visualization. Each iteration randomly assigned resistance phenotypes across antibiotics within the constraints of the actual data (same number of isolates and antibiotic variables), allowing estimation of an expected MDR distribution under random conditions. This served as a baseline to assess whether the observed MDR prevalence was likely driven by non-random, external factors such as antibiotic usage patterns.

All analyses were conducted using open-source R packages to ensure full transparency and reproducibility. Antibiotic susceptibility profiles (antibiograms) were compiled from laboratory results and visualized using Microsoft Excel (Figures 6, 7).

## 5 Conclusion

This study provides a comprehensive overview of AMR profiles in *Enterococcus* spp. isolates isolated from domestic pigeons in Hungary during 2022. The high prevalence of MDR *Enterococcus* isolates (62.3%) and the limited presence of XDR isolates (3.8%) reflect a concerning trend in the development of antimicrobial resistance in pigeon populations. This pattern highlights the need for regular and comprehensive monitoring of antimicrobial susceptibility in marginal poultry species. In particular, the detection of high resistance rates to tylosin, florfenicol, and enrofloxacin underlines the importance of prudent antibiotic use and improved antimicrobial stewardship practices. Given the potential role of pigeons in disseminating resistant bacteria across regions — especially through homing and racing activities — a One Health-based approach is essential to assess and mitigate the broader public health risks. These findings support the urgent need for targeted surveillance programs and stricter regulation of antibiotic usage within this under-monitored sector of poultry farming.

The MIC data further support these findings, highlighting that imipenem and neomycin retained efficacy against ≥90% of the isolates. Network and cluster analyses revealed co-resistance patterns, suggesting selective pressures due to common use or cross-resistance mechanisms, particularly involving tylosin, florfenicol, and enrofloxacin. Decision tree modeling identified key predictors of MDR, underscoring the diagnostic and preventive value of resistance profiling. Monte Carlo simulations confirmed the robustness of the observed MDR prevalence and its potential stability across the broader population.

The comparative analysis with human *E. faecalis* and *E. faecium* resistance data underlines the zoonotic and public health relevance of pigeon-derived isolates. The significant differences in resistance patterns among species further emphasize the need for species-specific surveillance frameworks.

Given the increasing role of wildlife, including pigeons, in the environmental dissemination of AMR, these findings underscore the importance of integrating avian monitoring into broader One Health AMR surveillance programs. Future research should focus on molecular characterization of resistance genes and explore horizontal gene transfer potential within and across host species.

Our findings reinforce the hypothesis that pigeons may serve as significant reservoirs for MDR *Enterococcus* isolates, posing a potential public health risk. The presence of such isolates in pigeon populations underscores the need for routine monitoring and responsible antibiotic stewardship in both veterinary and human medicine.

Cross-sectoral collaboration, increased awareness, and tighter regulatory oversight of antimicrobial use are essential for mitigating the spread of MDR pathogens. The control of AMR is a global challenge that demands a comprehensive, One Health–aligned approach to preserve the efficacy of antibiotics for future generations.

Proactive mitigation strategies, rational antimicrobial use in veterinary settings, and strengthened biosecurity measures will be essential to curbing the spread of resistant *Enterococcus* isolates and safeguarding the long-term efficacy of critically important antimicrobials in both human and animal health domains.

## Data Availability

The original contributions presented in the study are included in the article/Supplementary material, further inquiries can be directed to the corresponding author.
