# Supplementary material for: Antimicrobial susceptibility profiles of Enterococcus spp. isolates from domestic pigeons in Hungary in 2022
Source: Front Vet Sci. 2025 Oct 21;12:1642910. doi: 10.3389/fvets.2025.1642910 (PMC12584079; doi:10.3389/fvets.2025.1642910)
Supplement: Supplementary file 1 [file Table_1.docx]

Supplementary Material

**Supplementary Table 1** Species identification of *Enterococcus* isolates (*n*=53) isolated from pigeons using MALDI-TOF.

| **Number** | **MALDI-TOF** | **Log (score)** | **Antimicrobial resistance profile** |
| --- | --- | --- | --- |
| 1 | *Enterococcus faecium* | 2.41 | ENR |
| 2 | *Enterococcus faecium* | 2.85 | FLO |
| 3 | *Enterococcus faecalis* | 2.47 | AMX, AMC, FLO, TIL |
| 4 | *Enterococcus columbae* | 2.52 | AMX, AMC, TIL |
| 5 | *Enterococcus faecium* | 2.32 | AMX, AMC, FLO, TIL |
| 6 | *Enterococcus faecalis* | 2.37 | TIL |
| 7 | *Enterococcus faecalis* | 2.22 | AMX, TIL |
| 8 | *Enterococcus durans* | 2.31 | AMX, AMC, FLO, TIL |
| 9 | *Enterococcus faecium* | 2.47 | AMX, AMC, FLO, TIL |
| 10 | *Enterococcus faecium* | 2.35 | ^[[1]](#footnote-1)^ |
| 11 | *Enterococcus faecium* | 2.46 | ^[[2]](#footnote-2)^ |
| 12 | *Enterococcus faecalis* | 2.31 | AMX, AMC, FLO, TIL, ENR |
| 13 | *Enterococcus faecalis* | 2.55 | DOX, PSA |
| 14 | *Enterococcus faecium* | 2.22 | DOX, TIL |
| 15 | *Enterococcus durans* | 2.14 | FLO, TIL |
| 16 | *Enterococcus faecium* | 2.15 | AMX, DOX, FLO, TIL, ENR, PSA |
| 17 | *Enterococcus faecium* | 2.27 | TIL |
| 18 | *Enterococcus durans* | 2.59 | DOX, TIL, ENR, PSA |
| 19 | *Enterococcus durans* | 2.69 | TIL |
| 20 | *Enterococcus durans* | 2.59 | AMX, AMC, DOX, FLO, TIL, ENR, PSA |
| 21 | *Enterococcus columbae* | 2.14 | AMX, AMC, DOX, FLO, TIL, ENR, PSA |
| 22 | *Enterococcus faecium* | 2.13 | AMX, AMC, DOX, FLO, TIL, ENR, PSA |
| 23 | *Enterococcus faecalis* | 2.11 | AMX, AMC, DOX, FLO, TIL, PSA |
| 24 | *Enterococcus durans* | 2.87 | AMX, AMC, DOX, FLO, TIL, PSA, VAN |
| 25 | *Enterococcus hirae* | 2.55 | AMX, AMC, DOX, FLO, TIL, ENR |
| 26 | *Enterococcus durans* | 2.59 | FLO, TIL, ENR, VAN |
| 27 | *Enterococcus mundtii* | 2.37 | AMC, DOX, FLO, TIL, ENR |
| 28 | *Enterococcus faecalis* | 2.38 | AMC, FLO, TIL, ENR |
| 29 | *Enterococcus faecalis* | 2.38 | DOX, FLO, TIL, ENR |
| 30 | *Enterococcus gallinarum* | 2.25 | IMI, FLO, TIL, ENR, VAN |
| 31 | *Enterococcus faecalis* | 2.22 | IMI, DOX, FLO, TIL, ENR |
| 32 | *Enterococcus columbae* | 2.27 | DOX, FLO, TIL, ENR |
| 33 | *Enterococcus faecalis* | 2.39 | AMX, AMC, DOX, FLO, TIL, ENR |
| 34 | *Enterococcus faecalis* | 2.47 | AMX, AMC, DOX, FLO, TIL, ENR, VAN |
| 35 | *Enterococcus gallinarum* | 2.53 | AMX, DOX, FLO, TIL, ENR, PSA, VAN |
| 36 | *Enterococcus hirae* | 2.55 | AMC, DOX, FLO, TIL, ENR, PSA, VAN |
| 37 | *Enterococcus gallinarum* | 2.17 | AMC, FLO, TIL, ENR |
| 38 | *Enterococcus faecalis* | 2.19 | FLO, TIL, ENR |
| 39 | *Enterococcus faecalis* | 2.29 | AMX, DOX, FLO, TIL, ENR |
| 40 | *Enterococcus faecium* | 2.44 | ^[[3]](#footnote-3)^ |
| 41 | *Enterococcus faecium* | 2.61 | DOX, TIL, ENR, PSA |
| 42 | *Enterococcus faecium* | 2.82 | TIL |
| 43 | *Enterococcus faecalis* | 2.77 | FLO, TIL |
| 44 | *Enterococcus columbae* | 2.91 | DOX, FLO, TIL, ENR |
| 45 | *Enterococcus faecalis* | 2.37 | ENR |
| 46 | *Enterococcus faecalis* | 2.48 | ENR |
| 47 | *Enterococcus gallinarum* | 2.55 | ENR |
| 48 | *Enterococcus faecalis* | 2.54 | AMX, DOX, TIL, ENR |
| 49 | *Enterococcus faecalis* | 2.36 | FLO, TIL |
| 50 | *Enterococcus faecalis* | 2.93 | AMX, DOX, FLO, TIL, PSA, VAN |
| 51 | *Enterococcus faecalis* | 2.82 | AMX, AMC, DOX, FLO, TIL, ENR, PSA, VAN |
| 52 | *Enterococcus columbae* | 2.71 | DOX, ENR |
| 53 | *Enterococcus faecalis* | 2.22 | TIL |

AMX - Amoxicillin, AMC - Amoxicillin-clavulanic acid, IMI - Imipenem, DOX - Doxycycline, FLO - Florfenicol, TYL - Tylosin, ENR - Enrofloxacin, PSA - Potentiated sulphonamide, VAN – Vancomycin

**Supplementary Table 2** Distribution of isolates by region and production type, the number of MDR isolates in parentheses.

| **Region** | **Homing** | **Meat** | **Ornamental** | **Total** |
| --- | --- | --- | --- | --- |
| Dél-Alföld | 7 (2) | 6 (4) | 14 (4) | **27 (10)** |
| Dél-Dunántúl | 1 (0) | 2 (2) | 0 (0) | **3 (2)** |
| Észak-Alföld | 1 (1) | 0 (0) | 0 (0) | **1 (1)** |
| Észak-Magyarország | 4 (3) | 0 (0) | 0 (0) | **4 (3)** |
| Közép-Dunántúl | 14 (14) | 0 (0) | 0 (0) | **14 (14)** |
| Közép-Magyarország | 0 (0) | 0 (0) | 2 (2) | **2 (2)** |
| Nyugat-Dunántúl | 2 (1) | 0 (0) | 0 (0) | **2 (1)** |
| **Total** | **29 (21)** | **8 (6)** | **16 (6)** | **53 (33)** |

**Supplementary Table 3** Distribution of isolates by region and age, the number of MDR isolates in parentheses.

| **Region** | **Shippet** | **Young** | **Adult** | **Breeding** | **Total** |
| --- | --- | --- | --- | --- | --- |
| Dél-Alföld | 7 (3) | 14 (2) | 2 (1) | 4 (4) | **27 (10)** |
| Dél-Dunántúl | 0 (0) | 2 (2) | 1 (0) | 0 (0) | **3 (2)** |
| Észak-Alföld | 0 (0) | 1 (1) | 0 (0) | 0 (0) | **1 (1)** |
| Észak-Magyarország | 0 (0) | 0 (0) | 4 (3) | 0 (0) | **4 (3)** |
| Közép-Dunántúl | 0 (0) | 3 (3) | 7 (7) | 4 (4) | **14 (14)** |
| Közép-Magyarország | 0 (0) | 0 (0) | 2 (2) | 0 (0) | **2 (2)** |
| Nyugat-Dunántúl | 0 (0) | 1 (0) | 0 (0) | 1 (1) | **2 (1)** |
| **Total** | **7 (3)** | **21 (8)** | **16 (13)** | **9 (9)** | **53 (33)** |

**Supplementary Table 4** Distribution of isolates by region and flock size, the number of MDR isolates in parentheses.

| **Region** | **1-50** | **51-100** | **101-500** | **<501** | **Total** |
| --- | --- | --- | --- | --- | --- |
| Dél-Alföld | 7 (2) | 11 (1) | 3 (3) | 6 (4) | **27 (10)** |
| Dél-Dunántúl | 0 (0) | 1 (0) | 2 (2) | 0 (0) | **3 (2)** |
| Észak-Alföld | 0 (0) | 0 (0) | 1 (1) | 0 (0) | **1 (1)** |
| Észak-Magyarország | 0 (0) | 1 (1) | 3 (2) | 0 (0) | **4 (3)** |
| Közép-Dunántúl | 0 (0) | 14 (14) | 0 (0) | 0 (0) | **14 (14)** |
| Közép-Magyarország | 0 (0) | 2 (2) | 0 (0) | 0 (0) | **2 (2)** |
| Nyugat-Dunántúl | 0 (0) | 2 (1) | 0 (0) | 0 (0) | **2 (1)** |
| **Total** | **7 (2)** | **31 (19)** | **9 (8)** | **6 (4)** | **53 (33)** |

1. Blank cell in the Antimicrobial resistance profile column indicate that no antimicrobial resistance was detected. [↑](#footnote-ref-1)
2. Blank cell in the Antimicrobial resistance profile column indicate that no antimicrobial resistance was detected. [↑](#footnote-ref-2)
3. Blank cell in the Antimicrobial resistance profile column indicate that no antimicrobial resistance was detected. [↑](#footnote-ref-3)
